# Longitudinal MRI in comparison to low-dose CT for follow-up of incidental pulmonary nodules in patients with COPD—a nationwide multicenter trial

**DOI:** 10.1007/s00330-025-11567-4

**Published:** 2025-04-13

**Authors:** Lin Zhu, Qian Li, Oyunbileg von Stackelberg, Simon M. F. Triphan, Jürgen Biederer, Oliver Weinheimer, Monika Eichinger, Claus F. Vogelmeier, Rudolf A. Jörres, Hans-Ulrich Kauczor, Claus P. Heußel, Bertram J. Jobst, Hong Yu, Mark O. Wielpütz, for the COSYCONET study group

**Affiliations:** 1 Department of Radiology, Shanghai Chest Hospital, School of Medicine, Shanghai Jiao Tong University, Shanghai, China; 2 Department of Diagnostic and Interventional Radiology, University Hospital of Heidelberg, Heidelberg, Germany; 3 Translational Lung Research Center Heidelberg (TLRC), German Center for Lung Research (DZL), Heidelberg, Germany; 4 Department of Diagnostic and Interventional Radiology with Nuclear Medicine, Thoraxklinik at the University Hospital of Heidelberg, Heidelberg, Germany; 5 Departments of Radiology, Union Hospital, Tongji Medical College, Huazhong University of Science and Technology, Wuhan, China; 6 Faculty of Medicine, University of Latvia, Riga, Latvia; 7 Faculty of Medicine, Christian-Albrechts-Universität zu Kiel, Kiel, Germany; 8 Department of Medicine, Pulmonary and Critical Care Medicine, Philipps-University of Marburg (UMR), Marburg, Germany; 9 Institute and Outpatient Clinic for Occupational, Social and Environmental Medicine, University Hospital, Ludwig Maximilians University (LMU) Munich, Comprehensive Pneumology Center Munich (CPC-M), Munich, Germany; 10 Department of Diagnostic Radiology and Neuroradiology, University Medicine Greifswald, Greifswald, Germany

**Keywords:** Pulmonary nodules, Chronic obstructive pulmonary disease, Low-dose computed tomography, Magnetic resonance imaging, Longitudinal management

## Abstract

**Purpose:**

This multicenter trial was conducted to evaluate MRI for the longitudinal management of incidental pulmonary nodules in heavy smokers.

**Materials and methods:**

239 participants (63.9 ± 8.4 years, 43–82 years) at risk of or with COPD GOLDI-IV from 16 centers prospectively underwent two rounds of same-day low-dose computed tomography (LDCT1&2) and MRI1&2 at an interval of three years in the nationwide COSYCONET trial. All exams were independently assessed for incidental pulmonary nodules in a standardized fashion by two blinded readers, incl. axis measurements and Lung-RADS categorization, with consensual LDCT results serving as the standard of reference. A change in diameter ≥ 2 mm was rated as progress. 11 patients underwent surgery for suspicious nodules after the first round.

**Results:**

Two hundred twenty-four of two hundred forty nodules (93.3%) persisted from LDCT1 to LDCT2, with a sensitivity of MRI2 of 82.8% and 81.5% for readers 1 and 2, respectively. Agreement in Lung-RADS categories between LDCT2 and MRI2 was substantial in per-nodule (κ = 0.62–0.70) and excellent in a per-patient (κ = 0.86–0.88) approach for both readers, respectively. Concordance between LDCT2 and MRI2 for growth was excellent to almost perfect (κ = 0.88–1.0). The accuracy of LDCT1 and MRI1 for lung cancer was 87.5%. Lung-RADS ≥ 3 category on MRI1 had higher accuracy for predicting progress (23.1% and 21.4%, respectively) than LDCT1 (15.8%).

**Conclusion:**

Compared to LDCT, MRI shows similar capabilities for the longitudinal evaluation of incidental nodules in heavy smokers. Decision-making for nodule management guided by Lung-RADS seems feasible based on longitudinal MRI.

**Key Points:**

***Question*** *Can MRI serve as an alternative to low-dose CT (LDCT) for the longitudinal management of pulmonary nodules in heavy smokers, addressing concerns over radiation exposure*?

***Findings*** *MRI demonstrated substantial agreement with LDCT in detecting nodule growth, accurately categorizing Lung-RADS, and comparable accuracy in identifying malignancy over a three-year follow-up*.

***Clinical relevance*** *Longitudinal MRI demonstrates high consistency with LDCT in assessing the growth of incidental pulmonary nodules and categorizing per-patient Lung-RADS, offering a reliable, radiation-free alternative for monitoring and early malignancy detection in high-risk populations*.

**Graphical Abstract:**

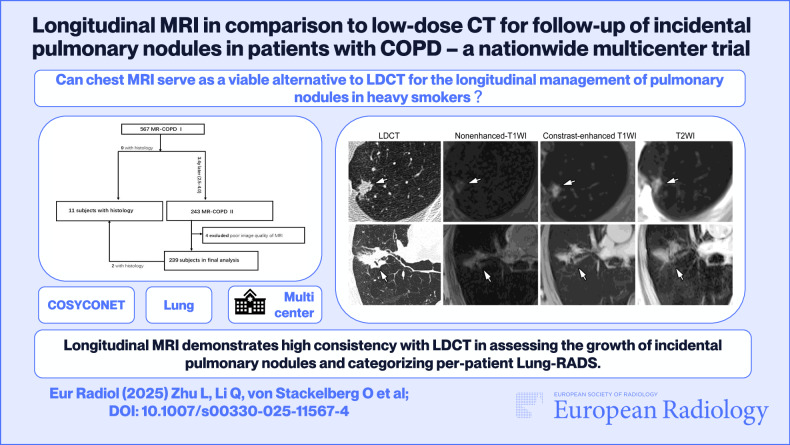

## Introduction

Low-dose CT (LDCT) lung cancer screening has been shown to reduce lung cancer mortality [1–4]. Meanwhile, ionizing radiation from the repetitive application of CT in long-term screening programs increases individual dose accumulation and the risk of developing cancer, which may counteract the positive effects of screening on cancer-specific mortality [5, 6]. Annual LDCT screening was estimated to add the additional risk of individual participants developing lung cancer with an upper limit of 5.5% [7]. Chronic obstructive pulmonary disease (COPD) and airflow obstruction are additional risk factors for developing lung cancer independent of smoking habits. Morpho-functional chest magnetic resonance imaging (MRI) has recently been introduced as a novel modality for the assessment of the severity of muco-obstructive lung diseases such as COPD and cystic fibrosis [8–12]. Further evidence also indicates that chest MRI may be suitable as an alternative to LDCT for the detection and management of pulmonary nodules, especially in COPD patients [13]. MRI may even be more efficient than LDCT as a lung cancer screening tool because of a lower number of false positive findings and a relatively higher sensitivity for malignant than for benign lesions [14–16]. In a recent study comparing morpho-functional MRI with same-day LDCT for the detection and characterization of incidental pulmonary nodules in subjects with COPD in 16 participating centers in the prospective nationwide German multicenter trial ‘COPD and SYstemic consequences-COmorbidities NETwork’ (COSYCONET), we could show that MRI with a standardized morpho-functional protocol has moderate sensitivity for incidental pulmonary nodules in COPD patients. Moreover, MRI seemed to be suitable for management decisions based on the Lung CT screening and reporting system (Lung-RADS) in a per-patient approach in reference to LDCT [17]. However, no longitudinal data on the follow-up of pulmonary nodules using MRI to assess changes in lung nodule size and characteristics are available to date. This, however, is a prerequisite to potentially employing MRI for the management of pulmonary nodules. Therefore, the objective of the present study was to follow up on incidental pulmonary nodules in patients with or at risk of COPD in the COSYCONET cohort using the same morpho-functional MRI protocol in comparison to same-day LDCT approximately three years after the first round of imaging. This allowed for re-visiting the prognostic properties of MRI at the first round of imaging, including respective Lung-RADS classifications were revisited in view of the clinical outcome after three years.

## Materials and methods

### Study cohort

**Fig. 1:**
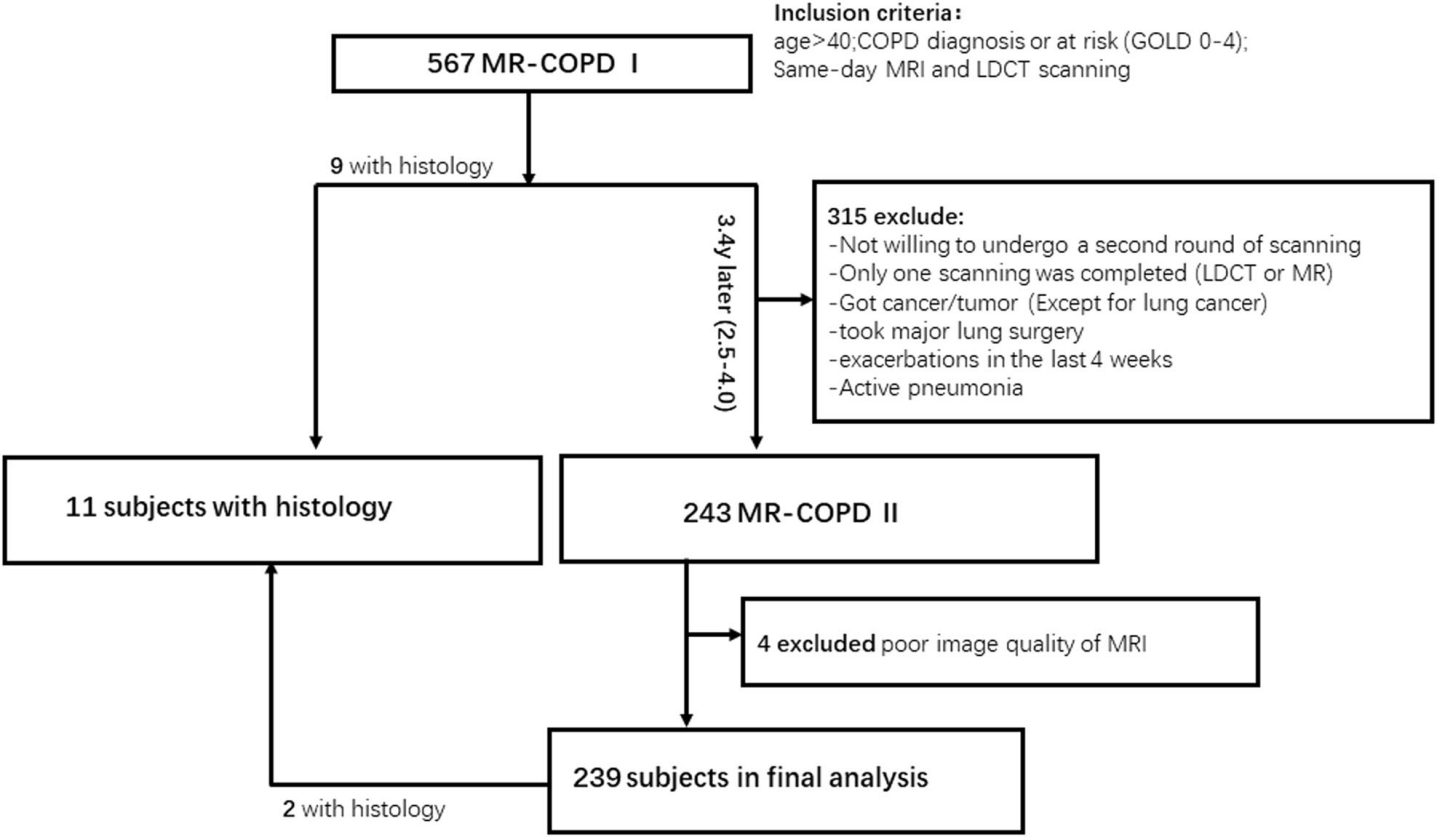
Study flow chart

The present study is part of the prospective imaging substudy “Image-Based Structural and Functional Phenotyping of the COSYCONET Cohort Using MRI and CT” [MR-COPD, NCTclinicaltrials.gov identifier NCT02629432] of the longitudinal multicenter COSYCONET cohort study (NCT01245933) across 31 German centers. All subjects signed an informed consent protocol. The study adhered to the principles of the Declaration of Helsinki and received approval from the ethics committee of Heidelberg University (S-400/2016). Of 2.741 patients in COSYCONET, 567 subjects at 16 centers were imaged in the first round, and 239 subjects (42.2%) (baseline mean age 63.9 ± 8.4 years, range 43–82 yrs) underwent a second imaging round with MRI and same-day LDCT after about three years (mean follow-up 3.4 ± 0.6 years, range 2.5–4.0 yrs) between February 2014 and December 2019 [17]. Eleven patients (with 13 nodules or masses, respectively) underwent invasive procedures for a suspicious finding, providing histological proof of malignancy (Fig. 1). The characteristics of the participants and nodules are shown in Table 1. A detailed description is provided in the online supplement.

**Table 1:** Participant characteristics

Age	
Male (*n* = 152)	65.7 ± 8.0 (47–82)
Female (*n* = 87)	63.5 ± 8.4 (43–78)
GOLD stage (*n*)	
At risk	34
I	53
II	86
III	55
IV	11
Participants underwent surgery/ biopsy (*n*)	11
Participants with new nodules at LDCT2 (*n*)	8
No. of participants with nodules (*n*)	86
No. of nodules present throughout LDCT1 and 2	224
Progressed	19
Stable	181
Regressed/disappeared	24/16
No. of nodules present throughout MRI1 and 2 (Reader1; Reader2)	180; 179
Progressed	13/14
Stable	151/146
Regressed/disappeared	16/17; 19/17

Data are presented as means ± SDs, with ranges in parentheses, or numbers of participants or nodules

*GOLD* global initiative for chronic obstructive lung disease criteria, *LDCT* low-dose computed tomography

### Morpho-functional chest MRI

All subjects underwent 1.5-T or 3.0-T MRI encompassing major vendors (Siemens Healthineers; General Electric; Philips) using a standardized chest MRI protocol identical to the first imaging round and repeatedly validated by phantom measurements as described previously [12, 17–19]. Between the two imaging sessions, none of the centers exchanged MRI scanner hardware. In brief, morpho-functional MRI included nonenhanced and contrast-enhanced T1-weighted, and T2-weighted sequences in different breath holds and orientations. The full morpho-functional MRI protocol involved i.v. contrast injection of Gadobutrol (Bayer Vital GmbH, 0.05 mmol/kg) at a rate of 5 mL/s followed by a saline flush (30 mL NaCl) as previously described for perfusion imaging, which was not further assessed for the purpose of the present study, but allowed for a post-contrast T1 GRE acquisition (Supplemental Table S1) [17, 19]. The acquisition time for the whole morpho-functional protocol approximated 30 min. Specifically, the three sequences studied separately for the present study encompassed measurement times of 16 s, 17–19 s, and 16–35 s for transversal 3D nonenhanced and contrast-enhanced T1-weighted, as well as T2-weighted sequence acquisitions in the inspiratory state, respectively (Supplemental Table S1). The slice thickness of the transversal 3D nonenhanced and contrast-enhanced T1-weighted, and T2-weighted sequences was 4 mm, 4 mm, and 8 mm, respectively [17–20].

### Chest low-dose computed tomography

All subjects underwent standardized non-enhanced LDCT encompassing major vendors (Siemens Healthineers; General Electric; Philips) identical to the protocol of the first imaging round, and validated by repeated phantom measurements as previously described [17, 21]. Between the two imaging sessions, none of the centers exchanged CT scanner hardware. CT images were reconstructed at 0.625–1.0 mm contiguous slice thickness using smooth and edge-enhancing algorithms (B70f/LUNG/L and B30f/SOFT/B, referring to generic names of Siemens/General Electric/Philips, respectively). The maximum effective dose of paired inspiratory and expiratory LDCT was kept under 3.5 mSv [17].

### Image evaluation

Baseline and follow-up LDCT and MRI (LDCT1 and LDCT2, as well as MRI1 and MRI2, respectively) were assessed independently and strictly blinded to the other timepoint and modality, as well as clinical data, by two radiologists (L.Z. with 3 and Q.L. with 7 years of experience, respectively) with the method and workstation described previously (Supplemental Fig. S1) [17]. In brief, nodules larger than 3 mm were recorded as stated previously, and the frequency distribution of the size of included nodules (average of long and short axis) in two rounds of analyses is shown in Supplemental Fig. S2 [13, 15, 22]. The following parameters were evaluated for MRI and LDCT alike: Longest axial diameter and perpendicular short-axis diameter, attenuation (categories solid, part-solid and purely ground-glass opacity [GGO]), morphological characteristics (calcification, fat, spiculation, lobulation, cavitation/necrosis), location (peripheral if ≤ 5 mm from visceral pleura, non-peripheral > 5 mm), and proximity to fissures (intra-fissural when in contact with the fissure and perifissural if ≤ 5 mm from fissure) [22–25]. The relative percentage of GGO components in part-solid nodules was assessed in four categories: 0–25%, 26–50%, 51–75%, and 76–100% [15, 17].

For a second read, the observers were given access to baseline LDCT1 and MRI1 measurements, in order to assess changes in diameter and for the final Lung-RADS v2022 category for LDCT2 and MRI2 by direct comparison to LDCT1 and MRI1, respectively. Based on the change in the average diameter of long and short axis from LDCT1 to LDCT2 as reference, nodules were divided into four categories: progress if increase > 2 mm, regress if decrease > 2 mm, disappearance, or stable within ±2 mm [24, 25]. Change of morphological characteristics (spiculation, cavitation/necrosis, lobulation) was classified in three categories: newly appeared, disappeared, or stable from LDCT1/MRI1 to LDCT2/MRI2. Axis measurements on LDCT1 and LDCT2, respectively, were averaged for both readers, and the variables regarding detection and categorization based on LDCT1 and LDCT2 were re-evaluated in consensus by the two readers where initial ratings were different, in order to establish a consensus for LDCT1 and LDCT2 as the standard of reference.

### Statistical analyses

Statistics were performed using RStudio v1.3.1093 (RStudio Inc.) and SPSS 22.0 (SPSS Inc.). Continuous data are presented as mean ± SD, and comparisons were performed with Student’s unpaired *t*-tests or paired *t*-tests and the non-parametric Wilcoxon–Mann–Whitney test or Wilcoxon Signed Rank test as appropriate. Categorical variables were analyzed with Pearson’s chi-squared and Fisher’s exact test. We evaluated correlations using Pearson’s correlation coefficient (*r*). LDCT consensual results served as the standard of reference for calculating the sensitivity, specificity, and positive predictive value (PPV) of MRI1 and MRI2, respectively. Inter-method agreements on incidental nodule follow-up were assessed with Cohen’s weighted kappa [26, 27]. To compare differences in axis measurements between the two modalities and readers, respectively, the method of Bland and Altman was used [27]. *p* < 0.05 was considered statistically significant.

## Results

### Longitudinal MRI for the evaluation of nodule growth

**Fig. 2:**
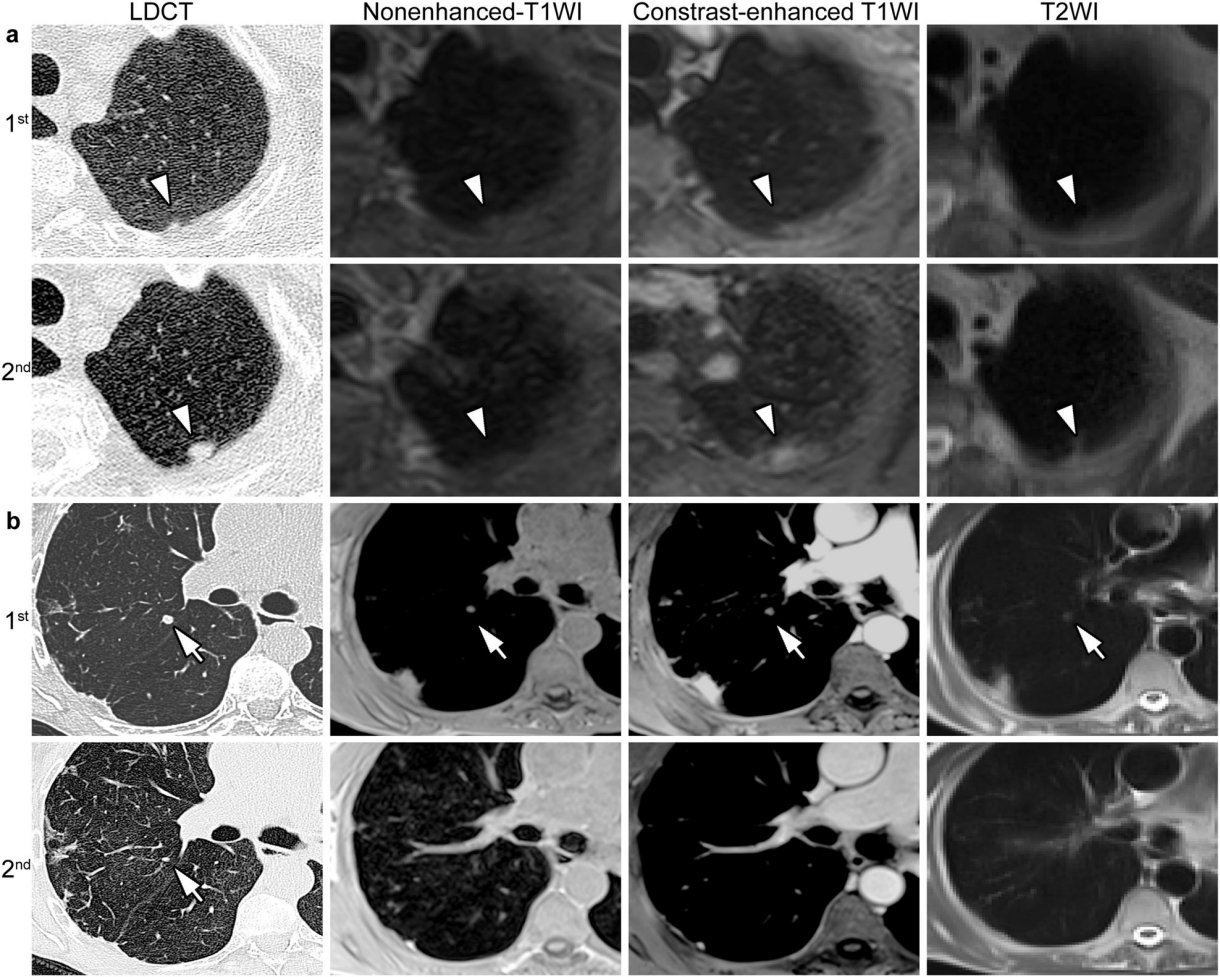
Representative images of progressed and regressed incidental nodules at morpho-functional MRI and LDCT. **a** A solid pulmonary nodule beside the visceral pleura showed significant growth on the second examination (second row, 2018) compared to the first examination (first row, 2015). The growth can be best displayed on the contrast-enhanced T1-weighted MR images (third column) than the nonenhanced (second column) and T2-weighted images (fourth column). **b** A solid nodule adjacent to the interlobar pleura was significantly shrunk on the second examination (fourth row) compared to the first round examination (third row). The shrunk nodules on the second imaging round were not displayed on the three MRI sequences

**Fig. 3:**
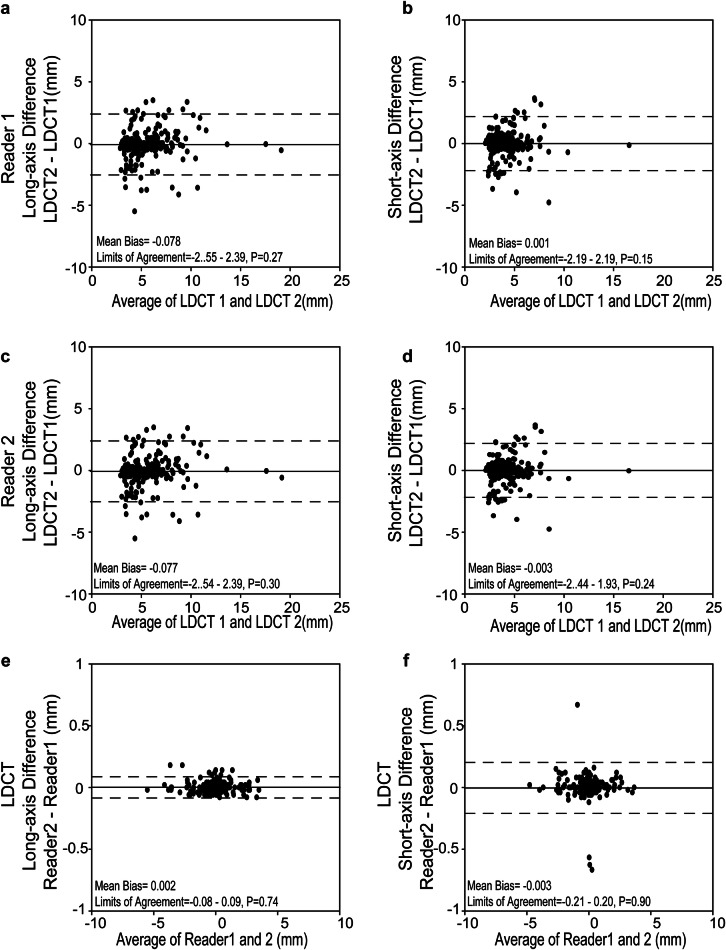
Longitudinal long- and short-axis diameter measurements of incidental nodules on LDCT for two imaging rounds. Comparison of the long- (**a**, **c**) and short-axis (**b**, **d**) diameter of incidental nodules for LDCT1 vs LDCT2 for Reader 1 (**a**, **b**) and Reader 2 (**c**, **d**), respectively. Inter-reader agreement for the longitudinal difference in long- (**e**) and short-axis (**f**) diameter measurements from LDCT1 to LDCT2. Dashed lines denote 95% confidence interval, and solid lines the mean. Mean bias and 95% CI are given at the bottom of each image, including *p*-values

Of the 240 nodules detected by LDCT1 in consensus by the two readers, 224 (93.3%) nodules persisted on LDCT2, and 16 (6.7%) nodules disappeared. Eight patients showed 14 newly developed nodules, amounting to a total of 238 nodules present on LDCT2 (Table 2). Nineteen nodules progressed in size from LDCT1 to LDCT2, 181 were stable, and 24 regressed in size (Fig. 2). Inter-reader agreement for growth categories using LDCT was perfect (κ = 0.99, 95% CI: 0.96–1.0). The measurements of the long- and short-axis diameter of persisting nodules revealed similar mean differences of −0.1 ± 1.3 mm (*p* = 0.27) and 0.0 ± 1.1 mm (*p* = 0.15) from LDCT1 to LDCT2 for reader 1, and −0.1 ± 1.3 mm (*p* = 0.30) and −0.0 ± 1.1 mm (*p* = 0.24) for reader 2, respectively. There was no statistically significant difference between the readers (*p* = 0.74–0.90) (Fig. 3).

**Table 2:** Diagnostic performance of contrast-enhanced T1-weighted MRI for incidental pulmonary nodules

			1st round						2nd round					
			LDCT	MRI					LDCT	MRI				
			Consensus, *n*	True positive, *n*	False negative, *n*	False positive, *n*	sensitivity	PPV	Consensus, *n*	True positive, *n*	False negative, *n*	False positive, *n*	Sensitivity	PPV
Reader 1	Solid	< 6 mm	186	156	30	6	83.9%	96.3%	184	150	34	0	81.5%	100.0%
		6–8 mm	28	25	3	1	89.3%	96.2%	33	31	2	1	93.9%	96.9%
		8–15 mm	13	12	1	0	92.3%	100.0%	12	11	1	0	91.7%	100.0%
		≥ 15 mm	1	1	0	0	100.0%	100.0%	2	2	0	0	100.0%	100.0%
	Part-solid	< 6 mm	4	4	0	0	100.0%	100.0%	2	2	0	0	100.0%	100.0%
		≥ 6 mm	2	1	1	1	50.0%	50.0%	0	0	0	0	NA	NA
	GGO	< 30 mm	6	1	5	0	16.7%	100.0%	5	1	4	0	20.0%	100.0%
Reader 2	Solid	< 6 mm	186	155	31	3	83.3%	98.1%	184	148	36	2	80.4%	98.7%
		6–8 mm	28	25	3	2	89.3%	92.6%	33	30	3	3	90.9%	90.9%
		8–15 mm	13	12	1	0	92.3%	100.0%	12	11	1	1	91.7%	91.7%
		≥ 15 mm	1	1	0	0	100.0%	100.0%	2	2	0	0	100.0%	100.0%
	Part-solid	< 6 mm	4	4	0	0	100.0%	100.0%	2	2	0	0	100.0%	100.0%
		≥ 6 mm	2	1	1	1	50.0%	50.0%	0	0	0	0	NA	NA
	GGO	< 30 mm	6	1	5	0	16.7%	100.0%	5	1	4	0	20.0%	100.0%

*GGO* ground-glass opacity, *LDCT* low-dose computed tomography, *MRI* magnetic resonance imaging, *PPV* positive predictive value, *n* number of nodules

**Fig. 4:**
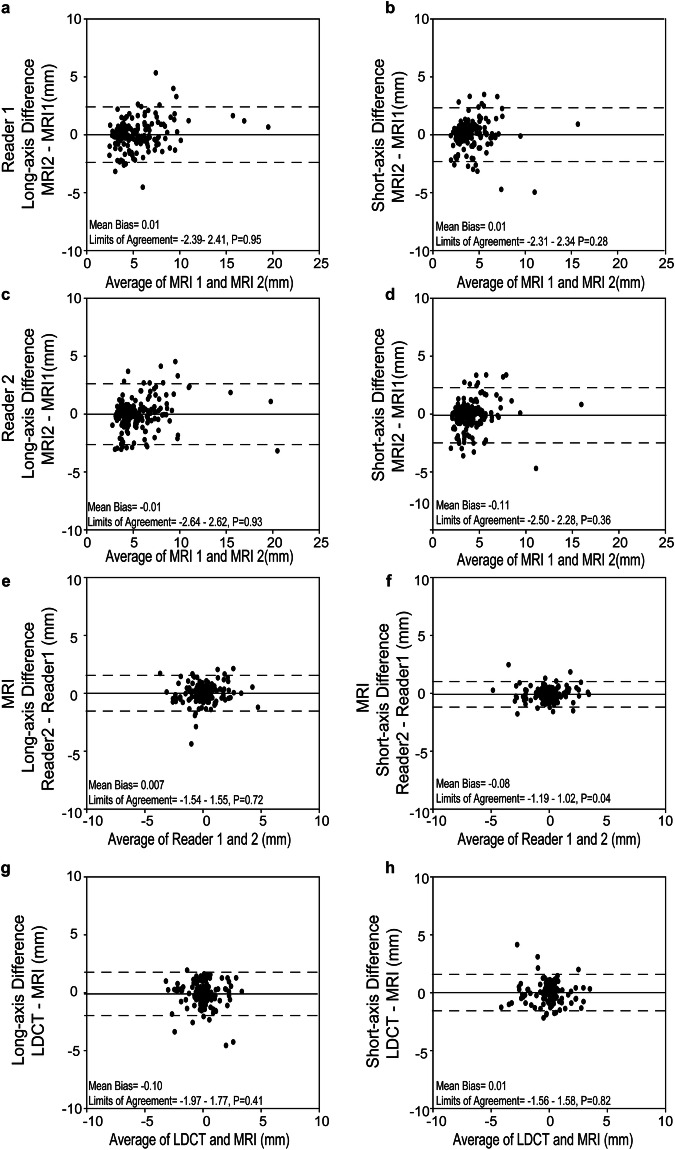
Longitudinal long- and short-axis diameter measurements of incidental nodules on MRI for two imaging rounds. Comparison of the long- (**a**, **c**) and short-axis (**b**, **d**) diameter of incidental nodules for MRI1 vs MRI2 for Reader 1 (**a**, **b**) and Reader 2 (**c**, **d**), respectively. Inter-reader agreement for the longitudinal change in long- (**e**) and short-axis (**f**) diameter measurements from MRI1 to MRI2. Inter-methods agreement for the average longitudinal change in long- (**g**) and short-axis (**h**) diameter measurements by two readers from MRI1 to MRI2 vs LDCT1 to LDCT2 as the standard of reference. Dashed lines denote 95% confidence interval, and solid lines the mean. Mean bias and 95% CI are given at the bottom of each image, including *p*-values

At MRI2, 197 nodules (82.8%) and 194 nodules (81.5%) out of 238 nodules on LDCT2 were detected by reader 1 and reader 2, respectively (Table 2). Regarding growth, 189 and 186 nodules were categorized identically using MRI compared to LDCT consensus by reader 1 and reader 2, respectively (κ = 0.90, CI: 0.83–0.97 and κ = 0.88, CI: 0.81–0.95) (Table 3). The measurements of the long- and short-axis diameter of persisting nodules on contrast enhanced T1-weighted MRI2 revealed mean differences of 0.0 mm ± 1.2 (*p* = 0.95) and −0.0 mm ± 1.3 (*p* = 0.93) from MRI1 to MRI2 for reader 1, and 0.0 mm ± 1.2 (*p* = 0.28) and −0.1 mm ± 1.2 (*p* = 0.36) for reader 2, respectively (Fig. 4). There was minimal to no statistically significant difference between both readers measuring long- and short-axis diameter changes on MRI (*p* = 0.04–0.72). There was no difference in assessing consensual diameter changes of long or short axis with MRI compared to LDCT (*p* = 0.41–0.82), and measurements of the two modalities were highly correlated for both readers (*r* = 0.63–0.74, *p* < 0.01, Supplemental Fig. S3). Measurements performed on T2 FSE with Half Fourier acquisition and balanced steady-state free-precession acquisitions are shown in the online supplement (Supplemental Table S2).

**Table 3:** Contingency table of growth categories for nodules detected at MRI 2 by reader 1 and reader 2 in comparison to LDCT2

Reader 1						Reader 2				
MRI	LDCT					LDCT				
All	Progressed	Stable	Regressed/disappear	Total	κ (95% CI)	Progressed	Stable	Regressed/disappear	Total	κ (95% CI)
Progressed	11	2	0	13	0.90 (0.83–0.97)	12	2	0	14	0.88 (0.81–0.95)
Stable	1	146	4	151		0	142	4	146	
Regressed/disappear	0	1	32	33		0	4	32	36	
Total	12	149	36	197		12	148	36	196	
Solid					0.89 (0.82–0.97)					0.89 (0.82–0.97)
Progressed	11	2	0	13		12	2	0	14	
Stable	1	144	4	148		0	140	4	144	
Regressed/disappear	0	1	28	29		0	4	28	32	
Total	12	147	32	191		12	146	32	190	
Non-solid					1.00 (1.00–1.00)					1.00 (1.00–1.00)
Progressed	0	0	0	0		0	0	0	0	
Stable	0	2	0	2		0	2	0	2	
Regressed/disappear	0	0	4	4		0	0	4	4	
Total	0	2	4	6		0	2	4	6	

Please note that nodules not detected at MRI are not included in the analysis. Non-solid nodules include GGO and part-solid nodules

*LDCT* low-dose computed tomography, *MRI* magnetic resonance imaging

### Longitudinal MRI for the evaluation of nodule morphology

The detection sensitivity of morphological features, including spiculation, cavitation/necrosis, and lobulation on MRI2 was similar to our previous first-round analysis, ranging from 36.8% to 71.4%. The additional assessments of proximity to fissures and peripheral location reached sensitivities ranging from 79.5% to 89.0% and 81.0% to 91.0% on MRI1 and MRI2, respectively (Supplemental Table S3). Both readers agreed substantially for spiculation and cavitation evolution (κ = 0.91–1.00, CI: 0.71–1.10, and κ = 0.84, CI: 0.51–1.17) and moderately for lobulation evolution (κ = 0.60–0.65, CI: 0.26–1.02) (Supplemental Tables S4 and S5).

### Longitudinal MRI for the assessment of Lung-RADS categories

Nodules were categorized according to Lung-RADS on LDCT2 in consensus, and strictly separately on MRI2 by both readers. For this assessment, LDCT1 and MRI1, respectively, were unblinded in order to allow for the inclusion of nodule growth and changes in morphology from LDCT1-2 and MRI1-2 into establishing the Lung-RADS category. In a per-nodule approach, the agreement for Lung-RADS at MRI2 with the reference was 0.70 (95% CI: 0.56–0.83) for reader 1 and 0.62 (95% CI: 0.48–0.77) for reader 2, respectively (Supplemental Table S6). In a per-patient approach, which relies only on the most suspicious nodule per patient and thus also includes patients without nodules, the agreement was 0.88 (95% CI: 0.82–0.94) for reader 1 and 0.86 (95% CI: 0.80–0.92) for reader 2, respectively (Table 4).

**Table 4:** Contingency table for Lung-RADS categorization with MRI2 and LDCT2 in a per-patient approach

MRI2	LDCT2								
	1	2	3	4 A	4B	4X	Total	κ	95% CI
Reader1								0.88	0.82–0.94
1	159	4	0	0	0	0	163		
2	2	52	1	1	0	0	56		
3	1	2	3	2	0	0	8		
4A	0	2	0	5	0	0	7		
4B	0	0	1	0	0	0	1		
4X	0	0	0	0	0	4	4		
Total	162	60	5	8	0	4	239		
Reader2								0.86	0.80–0.92
1	158	4	0	0	0	0	162		
2	3	53	1	2	0	0	59		
3	1	2	3	1	0	0	7		
4A	0	1	0	5	0	2	8		
4B	0	0	0	0	0	0	0		
4X	0	0	1	0	0	2	3		
Total	162	60	5	8	0	4	239		

Please note that the Lung-RADS categorization of the most suspicious nodule was rated as the categorization of patients with multiple nodules. Patients without nodules or only with nodules categorized as Lung-RADS 1 were rated as grade 1

*LDCT* low-dose computed tomography, *Lung-RADS* Lung CT screening and reporting system, *MRI* magnetic resonance imaging

### Prognostic capabilities of morpho-functional chest MRI for malignancy in incidental pulmonary nodules

Three of nineteen nodules that progressed on LDCT2 were categorized as Lung-RADS ≥ 3 on LDCT1 (accuracy 15.8%), and 200 of 221 nodules that were stable or regressed/disappeared were rated as Lung-RADS < 3 on LDCT1 (accuracy 84.6%). 3 of 13 and 14 nodules that progressed on MRI2 were categorized as Lung-RADS 3 or 4A by both reader 1 (accuracy 23.1%) and reader 2 (accuracy 21.4%) on MRI1, respectively. 164 of 184 and 163 of 182 nodules that were stable or regressed/disappeared on MRI2 were rated Lung-RADS < 3 by reader 1 (accuracy 89.1%) and reader 2 (accuracy 89.6%), respectively.

**Fig. 5:**
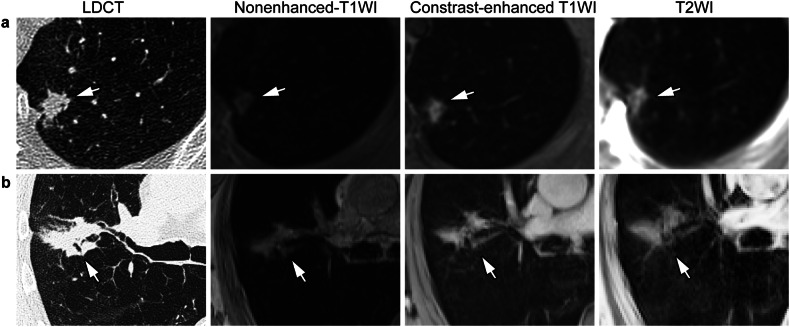
Examples of Lung-RADS classification of incidental nodules representing histologically proven lung cancer at LDCT and morpho-functional MRI. **a** A solid nodule with spiculated edges was classified as Lung-RADS grade 4A on both LDCT1 and MRI1. **b** A solid mass with lobulated edges and necrosis was classified as Lung-RADS grade 4X on both LDCT1 and MRI1

Nine patients after the first and two patients after the second round of imaging underwent procedures for histological proof of suspicious nodules, respectively, and were diagnosed with lung cancer (Fig. 5). Of note, three of the patients did not show any nodules on LDCT1 and MRI1, respectively, but developed an interval lung cancer before the second round of imaging. When all LDCT and all MRI from both imaging rounds were pooled, respectively, the diagnostic accuracy for malignancy in a per-patient approach was similar for LDCT and for MRI, as seven of eight patients with nodules (87.5%) were assigned Lung-RADS ≥ 3 (Supplemental Table S7).

## Discussion

To our knowledge, this is the first study employing longitudinal multicenter MRI in direct comparison to LDCT at two timepoints approximately 3 years apart to assess growth and changes in morphology of incidental pulmonary nodules in heavy smokers. We hereby demonstrate that morpho-functional MRI has moderate sensitivity for the detection of newly developed nodules > 3 mm, and excellent agreement with LDCT for the detection of nodule growth of > 2 mm. Further, agreement of longitudinal MRI with LDCT on Lung-RADS categories guiding nodule management, was excellent in a per-patient approach, and both modalities were equally accurate in detecting histologically proven lung cancer.

Sensitivity of MR2 for the detection of incidental pulmonary nodules was only moderate but slightly improved in comparison to our analysis of the first round of imaging [17]. Please note, that a relatively large number of small nodules between 4 mm and 5 mm were included (Fig. S2). The results align with previous studies by Meier-Schroers et al, which used a similar protocol without ultra-short echo time imaging, and showed poor to moderate sensitivity of MRI sequences to small nodules [28–30]. MRI’s sensitivity and specificity for detecting spiculation and cavitation/necrosis were consistent with our previous findings, and longitudinal MRI was consistent with LDCT in assessing morphological changes, which is also crucial for Lung-RADS categorization and outcome prediction of nodules [17]. Furthermore, this study indicates that enhanced T1-weighted imaging sequences offer superior detection and morphological assessment capabilities for nodules compared to non-enhanced T1WI and T2WI sequences. This indicated the possibility of employing this sole sequence in a nodule setting to effectively limit the time required for MRI-based lung screening and would allow for a high throughput.

The present data demonstrate an excellent agreement of MRI with LDCT in the evaluation of growth and diameter changes. MRI is highly correlated with the respective changes on LDCT. MRI was consistent with LDCT in measuring nodule diameter changes, as the systematic underestimation of diameters on MRI in comparison to LDCT is mitigated if intra-modality differences are assessed as in the present study [15, 17]. Since MRI equipment was kept strictly constant and phantom-controlled between the two rounds of examination, we were able to avoid measurement variation attributable to differences in MRI hardware, field strength, etc. [18]. In order to further assess the variability of nodule assessment with different MRI setups, further studies should be designed to scan pulmonary nodules with different MRI scanners on the same day, including artificial nodules in phantoms, for example [31]. Taken together, our data indicate that MRI may be reliable in assessing nodule growth.

Changes in nodule size and morphology can be taken into account when assigning Lung-RADS categories in the setting of follow-up imaging [24]. In the present study, we found a substantial inter-method agreement of MRI with LDCT at the second round of imaging, which was consistent with the results of the first round [17]. Inter-method agreement for Lung-RADS employing a per-patient approach was excellent in the present study. This may in part be due to the fact that MRI was able to correctly identify nodule growth > 2 mm according to the Lung-RADS recommendations, and thus may overrule the negative effect of underestimating the diameter in single timepoint assessments. Previous studies have implied that even changes as small as 1 mm in nodule diameter can be accurately detected across two rounds of follow-up MRI scans, which appeared to be consistent with the findings of the present study [28]. Related to the lower spatial resolution, MRI in general has the tendency to smooth the margins of nodules, but this effect appears to be weaker for larger nodules [15]. In the per-participant approach, the morphological margin characteristics of the most suspicious nodules, which are typically the largest nodules in each participant, can be more precisely identified through a two-round comparative analysis.

Remarkably, the study also demonstrated that MRI was consistent with LDCT in categorizing nodules into Lung-RADS ≥ 3 in 7 of 8 patients, which were then histologically proven to have lung cancer. Considering the relatively small sample size, the cancer rate was relatively high. Earlier population-based research indicated a 3.7–5.5% cancer rate in a screening setting [32]. The present study showed that 19 of 240 (7.9%) incidental nodules progressed during our longitudinal observation in the patients with or at risk of COPD background, and 40 of 240 (16.7%) regressed or disappeared on the other hand. These data are similar to lung cancer incidence (7.0–7.7%) in patients with impaired lung function observed in the German Lung Cancer Screening Intervention Study (LUSI) [33]. The observed high progression rate of incidental nodules in our study aligns with prior studies showing that both the presence and severity of emphysema independently increase lung cancer risk [34, 35]. In our cohort of 567 initial patients, 11 underwent histological verification, resulting in a lung cancer prevalence of 1.9% confirmed by histology. This rate is comparable, yet marginally higher than prior studies in smokers, which reported a prevalence of 1.1% to 1.4% [36]. Lung cancer screening employing MRI may help to further reduce risks attributable to screening examinations, and also to alleviate perceived risk due to radiation anxiety [37]. Further, the present study setting in a high-risk population could be disseminated to a wider clinical population examined with the intention to detect and characterize pulmonary nodules, e.g., in younger patients and children with cancer. Our data further supports that for incidental nodules found in chest MRI for other purposes, e.g., cardiac MRI, functional MRI in lung disease, etc., MRI may suffice as a technique to guide further nodule management without immediate need for a supplemental CT scan.

As a limitation and potential bias, only less than half of the patients from the first round were included in the second round. This was related to the main study design, which focused on COPD, and for which statistics considered it sufficient to limit the number of patients for the second round. Additionally, severely ill patients not willing to or capable of participating in the second round, and the high mortality of late-stage COPD, further reduced participation in the second round. Previous studies by Concatto et al and Usuda et al have demonstrated that the mean apparent diffusion coefficient (ADC) and Lesion-to-Spinal Cord Ratio (LSR) are higher in benign lesions and lower in lung cancer, with significant differences between the two. Additionally, T2 signal intensity ratios exhibited contrasting values. Specifically, the T2 contrast ratio in T2-weighted imaging (T2WI) was significantly lower in lung cancer compared to benign lesions, particularly in comparison to pulmonary abscesses [38, 39]. In light of these findings, the differences in signal characteristics of incidental pulmonary nodules among high-risk populations, as well as their progressive changes over time in individuals with lung cancer, warrant further investigation in future studies with an expanded sample size.

In conclusion, our study demonstrates the potential of standardized morpho-functional chest MRI for the longitudinal assessment and management of incidental pulmonary nodules in COPD patients in a multicenter setting. Longitudinal MRI appears to have a substantial accuracy in the detection of subtle pulmonary nodule changes and malignancy risk, surpassing single timepoint MRI assessments, approximating the capabilities of longitudinal LDCT. This highlights the potential capabilities of MRI in follow-up strategies for incidental pulmonary nodules in high-risk populations and warrants further investigation.

## Supplementary information

ELECTRONIC SUPPLEMENTARY MATERIAL

## Abbreviations

COPD: Chronic obstructive pulmonary disease
COSYCONET: COPD and systemic consequences-comorbidities network
GGO: Ground-glass opacity
GOLD: Global initiative for chronic obstructive lung disease
LDCT: Low-dose CT
Lung-RADS: Lung CT screening reporting and data system
