# Supplementary material for: Longitudinal MRI in comparison to low-dose CT for follow-up of incidental pulmonary nodules in patients with COPD—a nationwide multicenter trial
Source: Eur Radiol. 2025 Apr 13;35(10):6336–49. doi: 10.1007/s00330-025-11567-4 (PMC12417304; doi:10.1007/s00330-025-11567-4)
Supplement: Supplementary file 1 — ELECTRONIC SUPPLEMENTARY MATERIAL [file 330_2025_11567_MOESM1_ESM.pdf]

Longitudinal MRI in Comparison to low-dose CT for Follow-Up of Incidental Pulmonary Nodules in Patients with COPD – A Nationwide Multicenter Trial

ELECTRONIC SUPPLEMENTARY MATERIAL

Eur Radiol (2025) Zhu L, Li Q, von Stackelberg O, et al.

SUPPLEMENTAL MATERIALS AND METHODS

Study cohort

The present study was part of the prospective imaging substudy "Image-Based Structural and Functional Phenotyping of the COSYCONET Cohort Using MRI and CT" [MR-COPD, NCTclinicaltrials.gov identifier NCT02629432]) of the ongoing longitudinal multicenter COSYCONET cohort study (NCT01245933). All subjects signed an informed consent protocol. The study adhered to the principles of the Declaration of Helsinki and received approval from the ethics committee of Heidelberg University (S-400/2016). The in- and exclusion criteria of MR-COPD can be found elsewhere [1-3]. In brief, the population-based sample included patients aged 40 years and older with COPD diagnosed according to the Global Initiative for Chronic Obstructive Lung Disease (GOLD) criteria [4]. In addition, subjects with symptoms of COPD but no assignable GOLD category because of normal lung function (former 'GOLD 0') were included as subjects at risk [5; 6]. Exclusion criteria were previous major lung surgery, moderate to severe exacerbations within the last four weeks and active pneumonia. A total of 2.741 patients were recruited in 31 study centers throughout Germany for the COSYCONET trial, of which 567 subjects at 16 centers were prospectively recruited for the imaging substudy MR-COPD in our first round of imaging [1]. All patients were followed up after the imaging sessions by a standardized clinical protocol and interviews were reported elsewhere [2; 3]. 239 subjects (42.2%) (baseline mean age 63.9 ± 8.4 y, range 43 - 82 y) underwent a second imaging round with MRI and same-day LDCT after about three years (mean follow-up 3.4 ± 0.6 years, range 2.5 - 4.0 y) between February 2014 and December 2019 [1]. Eleven patients (with 13 nodules or masses, respectively) underwent invasive procedures for a suspicious finding, providing histological proof of malignancy (Figure 1). A detailed description of the study cohort is provided in Supplementary Note.

SUPPLEMENTAL REFERENCE

1 Li Q, Zhu L, von Stackelberg O et al (2023) MRI Compared with Low-Dose CT for Incidental Lung Nodule Detection in COPD: A Multicenter Trial. Radiol Cardiothorac Imaging 5:e220176

2 Jörres RA, Welte T, Bals R, Koch A, Schnoor M, Vogelmeier C (2010) [Systemic manifestations and comorbidities in patients with chronic obstructive pulmonary disease (COPD) and their effect on clinical state and course of the disease--an overview of the cohort study COSYCONET]. Dtsch Med Wochenschr 135:446-449

3 Karch A, Vogelmeier C, Welte T et al (2016) The German COPD cohort COSYCONET: Aims, methods and descriptive analysis of the study population at baseline. Respir Med 114:27-37

4 Vogelmeier CF, Criner GJ, Martinez FJ et al (2017) Global Strategy for the Diagnosis, Management, and Prevention of Chronic Obstructive Lung Disease 2017 Report. GOLD Executive Summary. Am J Respir Crit Care Med 195:557-582

5 Singh D, Agusti A, Anzueto A et al (2019) Global Strategy for the Diagnosis, Management, and Prevention of Chronic Obstructive Lung Disease: the GOLD science committee report 2019. Eur Respir J 53

6 Vestbo J, Hurd SS, Agustí AG et al (2013) Global strategy for the diagnosis, management, and prevention of chronic obstructive pulmonary disease: GOLD executive summary. Am J Respir Crit Care Med 187:347-365

SUPPELEMTAL TABLES

Supplemental Table S1. Standardized protocol for specific MRI sequences analyzed in the present study.

**1.5T MRI**												
**Sequence**	**Mode**	**Orientation**	**Respiration**	**Contrast**	**TR (ms)**	**TE (ms)**	**FoV (mm2)**	**slice thickness (mm)**	**Voxel size (mm2)**	**Matrix**	**PAT**	**Scan time (min:s)**
T1WI	3D	tra	insp	non-	3.29	1.61	400×300	4.0	1.25×1.25	320×240	2	0:16
T2WI	2D	tra	insp	contrast	500.0	27.0	450×366	8.0	1.41×1.41	320×260	2	0:35
T1WI FS	3D	tra	insp	contrast	3.29	1.61	400×300	4.0	1.25×1.25	320×240	2	0:17
**3.0T MRI**												
T1WI	3D	tra	insp	non-	3.00	0.87	450×338	4.0	1.80×1.80	384×512	2	0:16
T2WI	2D	tra	insp	contrast	452.0	28.0	450×338	8.0	1.80×1.80	192×256	2	0:16
T1WI FS	3D	tra	insp	contrast	3.00	0.87	450×338	4.0	1.80×1.80	384×512	2	0:19

Protocol designed on the 1.5T/3.0T Siemens platform, and then adapted to regionally available scanner hardware. tra = transversal, insp = inspiratory breathhold, TR = repetition time, TE = echo time, FoV = Field of view, PAT = parallelization factor, and FS= Fat saturation.

Supplemental Table S2. Diagnostic performance of non-enhanced T1- weighted and T2-weighted MRI for incidental pulmonary nodules.

			1st round				2nd round			
			**MRI T1WI**		**MRI T2WI**		**MRI T1WI**		**MRI T2WI**	
			Sensitivity	PPV	Sensitivity	PPV	Sensitivity	PPV	Sensitivity	PPV
**Reader1**	**Solid**	＜6mm	68.3%	95.5%	42.5%	95.2%	72.8%	98.5%	43.5%	97.6%
		6-8mm	78.6%	95.7%	46.4%	92.9%	84.8%	96.6%	42.4%	87.5%
		8-15mm	84.6%	100.0%	61.5%	100.0%	83.3%	100.0%	58.3%	100.0%
		≥15mm	100.0%	100.0%	100.0%	100.0%	100.0%	100.0%	100.0%	100.0%
	**Part-solid**	＜6mm	75.0%	75.0%	0.0%	0.0%	50.0%	50.0%	50.0%	50.0%
		≥6mm	50.0%	50.0%	50.0%	100.0%	NA	0.0%	NA	NA
	**GGO**	＜30mm	16.7%	100.0%	0.0%	NA	20.0%	100.0%	0.0%	NA
**Reader2**	**Solid**	＜6mm	66.7%	94.7%	41.4%	96.3%	71.7%	98.5%	42.4%	97.5%
		6-8mm	75.0%	95.5%	42.9%	92.3%	84.8%	96.6%	39.4%	92.9%
		8-15mm	84.6%	100.0%	61.5%	100.0%	83.3%	100.0%	58.3%	100.0%
		≥15mm	100.0%	100.0%	100.0%	100.0%	100.0%	100.0%	100.0%	100.0%
	**Part-solid**	＜6mm	50.0%	50.0%	0.0%	0.0%	50.0%	33.3%	0.0%	0.0%
		≥6mm	50.0%	50.0%	50.0%	100.0%	NA	NA	NA	NA
	**GGO**	＜30mm	16.7%	100.0%	0.0%	NA	20.0%	50.0%	0.0%	NA

Note.-GGO, ground-glass opacity; LDCT, low-dose computed tomography; MRI, magnetic resonance imaging; PPV, positive predictive value; n, number of nodules; T1WI, T1-weighted imaging; T2WI,T2-weighted imaging.

Supplemental Table S3. Morphological characteristics of incidental nodules detected by contrast-enhanced T1-weighted MRI in comparison to LDCT.

		1st round						2nd round					
		LDCT	MRI					LDCT	MRI				
		Consensus,n	True positive, n	False negative ,n	False positive, n	Sensitivity	Specificity	Consensus,n	True positive, n	False negative ,n	False positive, n	Sensitivity	Specificity
Reader 1	**Calcification**	13	0	13	0	0.0%	100.0%	20	0	20	0	0.0%	100.0%
	**Fat**	1	1	0	0	100.0%	100.0%	1	1	0	0	100.0%	100.0%
	**Spiculation**	10	4	6	2	40.0%	98.9%	14	6	8	2	42.9%	98.9%
	**Cavitation/ necrosis**	5	3	2	1	60.0%	99.5%	7	5	2	1	71.4%	99.5%
	**Lobulation**	19	8	12	6	40.0%	96.7%	18	9	9	11	50.0%	93.9%
	**Intra/perifissural**	44	35	9	3	79.5%	98.1%	42	34	8	2	81.0%	98.7%
	**Peripheral**	73	65	8	6	89.0%	95.3%	67	61	6	4	91.0%	96.9%
Reader 2	**Calcification**	13	0	13	0	0.0%	100.0%	20	0	20	0	0.0%	100.0%
	**Fat**	1	1	0	0	100.0%	100.0%	1	1	0	0	100.0%	100.0%
	**Spiculation**	10	4	6	3	40.0%	98.4%	14	6	8	2	42.9%	98.9%
	**Cavitation/ necrosis**	5	2	3	1	40.0%	99.5%	7	4	3	1	57.1%	99.5%
	**Lobulation**	19	7	12	6	36.8%	96.7%	18	8	10	10	44.4%	94.3%
	**Intra/perifissural**	44	35	9	2	79.5%	98.7%	42	34	8	1	81.0%	99.3%
	**Peripheral**	73	65	8	7	89.0%	94.4%	67	60	7	5	89.6%	96.1%

Note.-Please note that nodules not detected at MRI are not included in the analysis. LDCT, low-dose computed tomography; MRI, magnetic resonance imaging.

Supplemental Table S4. Contingency table of morphological evolution of nodule characteristics detected by contrast-enhanced T1-weighted MRI from MRI1 to MRI2 in comparison to LDCT.

	MRI1-MRI2	LDCT1-LDCT2				
		Newly appeared	Stable	Disappeared	Total	κ (95% CI)
Reader 1	**Spiculation**					
	Newly appeared	4	0	0	4	
	Stable	0	2	0	2	1.00 (1.00-1.00)
	Disappeared	0	0	2	2	
	Total	4	2	2	8	
	**Cavitation/necrosis**					
	Newly appeared	2	1	0	3	
	Stable	0	2	0	2	0.84 (0.52-1.16)
	Disappeared	0	0	1	1	
	Total	2	3	1	6	
	**Lobulation**					
	Newly appeared	2	1	0	3	
	Stable	0	5	1	6	0.60 (0.26-0.94)
	Disappeared	0	1	1	2	
	Total	2	7	2	11	
Reader 2	**Spiculation**					
	Newly appeared	3	1	0	4	
	Stable	0	2	0	2	0.91 (0.71-1.10)
	Disappeared	0	0	2	2	
	Total	3	3	2	8	
	**Cavitation/necrosis**					
	Newly appeared	2	1	0	3	
	Stable	0	1	0	1	0.84 (0.51-1.17)
	Disappeared	0	0	1	1	
	Total	2	2	1	5	
	**Lobulation**					
	Newly appeared	2	1	0	3	
	Stable	0	5	0	5	0.65 (0.28-1.02)
	Disappeared	0	1	1	2	
	Total	2	7	1	10	

Note.-Please note that nodules not detected at MRI are not included in the analysis. LDCT, low-dose computed tomography; MRI, magnetic resonance imaging.

Supplemental Table S5. Morphological characteristics of incidental nodules detected by non-enhanced T1- and T2-weighted MRI in comparison to LDCT.

		1st round				2nd round			
		**MRI T1WI**		**MRI T2WI**		**MRI T1WI**		**MRI T2WI**	
		Sensitivity	specificity	Sensitivity	specificity	Sensitivity	specificity	Sensitivity	specificity
**Reader1**	**Calcification**	0.0%	100.0%	0.0%	100.0%	0.0%	100.0%	0.0%	100.0%
	**Fat**	100.0%	100.0%	100.0%	100.0%	100.0%	100.0%	100.0%	100.0%
	**Spiculation**	20.0%	98.1%	10.0%	97.8%	28.6%	97.5%	28.6%	97.8%
	**Cavitation/ necrosis**	60.0%	98.8%	40.0%	99.0%	57.1%	99.4%	28.6%	100.0%
	**Lobulation**	36.8%	95.2%	10.5%	95.2%	38.9%	93.7%	27.8%	96.5%
	**Intra/perifissural**	77.3%	98.4%	78.0%	98.3%	73.8%	98.5%	69.0%	98.4%
	**Peripheral**	82.2%	92.5%	83.6%	79.3%	88.1%	96.3%	88.1%	86.5%
**Reader2**	**Calcification**	0.0%	100.0%	0.0%	100.0%	0.0%	100.0%	0.0%	100.0%
	**Fat**	100.0%	100.0%	100.0%	100.0%	100.0%	100.0%	100.0%	100.0%
	**Spiculation**	20.0%	98.0%	10.0%	97.8%	28.6%	97.5%	28.6%	97.7%
	**Cavitation/ necrosis**	40.0%	98.7%	20.0%	100.0%	42.9%	99.4%	28.6%	100.0%
	**Lobulation**	31.6%	95.1%	10.5%	95.0%	38.9%	94.2%	27.8%	97.6%
	**Intra/perifissural**	77.3%	98.3%	77.3%	96.4%	71.4%	99.2%	69.0%	98.3%
	**Peripheral**	82.2%	90.9%	84.9%	76.9%	88.1%	94.4%	91.0%	84.8%

Note.-Please note that nodules not detected at MRI are not included in the analysis. LDCT, low-dose computed tomography; MRI, magnetic resonance imaging; T1WI, T1-weighted imaging; T2WI,T2-weighted imaging; n, number of nodules.

Supplemental Table S6. Contingency table of Lung-RADS categories for nodules detected on MRI2 by reader 1 and reader 2 in comparison to LDCT2 in a per-nodule approach.

MRI2	LDCT2															
	**Reader 1**								**Reader 2**							
	1	2	3	4A	4B	4X	Total	κ (95% CI)	1	2	3	4A	4B	4X	Total	κ (95% CI)
All																
1	1	0	0	0	0	0	1		1	0	0	0	0	0	1	
2	8	153	4	1	0	1	167		9	149	3	3	0	1	165	
3	3	1	5	2	0	0	11	0.70 (0.56-0.83)	1	3	5	1	0	0	10	0.62(0.48-0.77)
4A	0	2	0	11	0	0	13		1	2	0	10	0	1	14	
4B	1	0	0	0	0	0	1		0	0	0	0	0	0	0	
4X	0	0	0	0	0	4	4		1	0	0	0	0	3	4	
Total	13	156	9	14	0	5	197		13	154	8	14	0	5	194	
Solid																
1	1	0	0	0	0	0	1		1	0	0	0	0	0	1	
2	8	150	4	1	0	1	164		9	146	3	3	0	1	162	
3	3	1	5	2	0	0	11	0.69 (0.56-0.83)	1	3	5	1	0	0	10	0.69 (0.56-0.83)
4A	0	2	0	11	0	0	13		1	2	0	10	0	1	14	
4B	1	0	0	0	0	0	1		0	0	0	0	0	0	0	
4X	0	0	0	0	0	4	4		1	0	0	0	0	3	4	
Total	13	153	9	14	0	5	194		13	151	8	14	0	5	191	
Non-solid																
1	0	0	0	0	0	0	0		0	0	0	0	0	0	0	
2	0	3	0	0	0	0	3	NA	0	3	0	0	0	0	3	NA
3	0	0	0	0	0	0	0		0	0	0	0	0	0	0	
4A	0	0	0	0	0	0	0		0	0	0	0	0	0	0	

4B	0	0	0	0	0	0	0	0	0	0	0	0	0	0
4X	0	0	0	0	0	0	0	0	0	0	0	0	0	0
Total	0	3	0	0	0	0	3	0	3	0	0	0	0	3

Note.-Please note that nodules not detected at MRI are not included in the analysis. Measurements performed on contrast enhanced T1-weighted MRI sequence. LDCT, low-dose computed tomography; Lung-RADS, Lung CT Screening & Reporting System; MRI, magnetic resonance imaging.

Supplemental Table S7. Lung-RADS grades of the 11 patients with histologically proven lung cancer assessed by LDCT1 and MRI1 in the first imaging round.

Patient No.	Lung-RADS category	
	LDCT1	MRI1 (Reader 1 / Reader 2)
1	4X	4X/4X
2	3	4X/4B
3	2	2/2
4	4B	4A/4B
5	4X	4X/4X
6	4A	4A/4A
7	4X	4X/4B
8	4B	4A/4A
9	1	1/1
10	1	1/1
11	1	1/1

Note.- Please note that the lung-RADS of patients were judged with a per-patient approach. The most suspicious nodule based on Lung-RADS was selected in subjects with multiple nodules to represent as the lung-RADS of patients and indicate further management. Measurements performed on contrast enhanced T1-weighted MRI sequence. LDCT, low-dose computed tomography; Lung-RADS, Lung CT Screening & Reporting System; MRI, magnetic resonance imaging.

SUPPLEMENTAL FIGURES

Patient	nodule	location		long axis (mm)	Short axis (mm)	benign fat	solid	Part solid				GGO	Morphological features			proximity to fissures	location	T1W-VIBE		T2W-HASTE		T1W-CE VIBE		Lung-RADS (T1W CE VIB)
								I	II	III	IV		cavitation	spiculation	lobulation			Yes	No	Yes	No	Yes	No	
xxx	Nx	☐ RUL<br>☐ LUL<br>☐ RML<br>☐ LLL<br>☐ RLL	1st			☐YES<br>☐NO	☐YES<br>☐NO	☐	☐	☐	☐	☐YES<br>☐NO	☐YES<br>☐NO	☐YES<br>☐NO	☐YES<br>☐NO	☐YES<br>☐NO	☐periphera<br>☐non-P							☐1 ☐4A<br>☐2 ☐4B<br>☐3 ☐4X
			2nd			☐YES<br>☐NO	☐YES<br>☐NO	☐	☐	☐	☐	☐YES<br>☐NO	☐YES<br>☐NO	☐YES<br>☐NO	☐YES<br>☐NO	☐YES<br>☐NO	☐periphera<br>☐non-P							☐1 ☐4A<br>☐2 ☐4B<br>☐3 ☐4X

Nx, the No.x nodule of the patient; I ,GGO component (0-20%); II , GGO component (26-50%); III , GGO component (51-75%); IV, GGO component (76-100%).

Supplemental Figure S1. Standardized questionnaire for the second round of nodule detection, measurement and categorization.

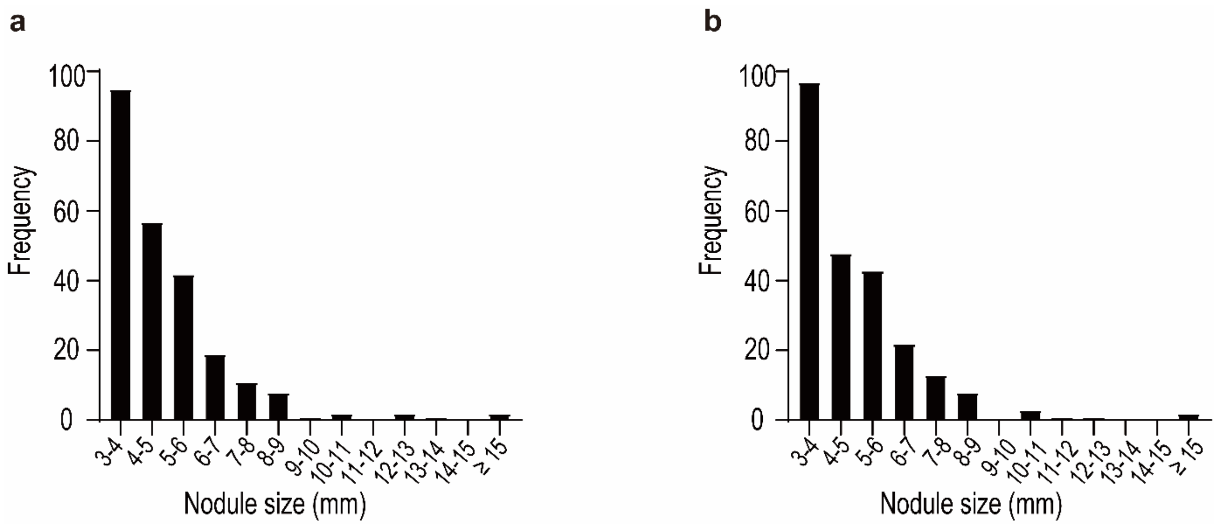

Supplemental Figure S2. Nodule size distribution. The histogram illustrates the frequency of average diameters of the long and short axis of included nodules on LDCT1 (a) and LDCT2 (b).

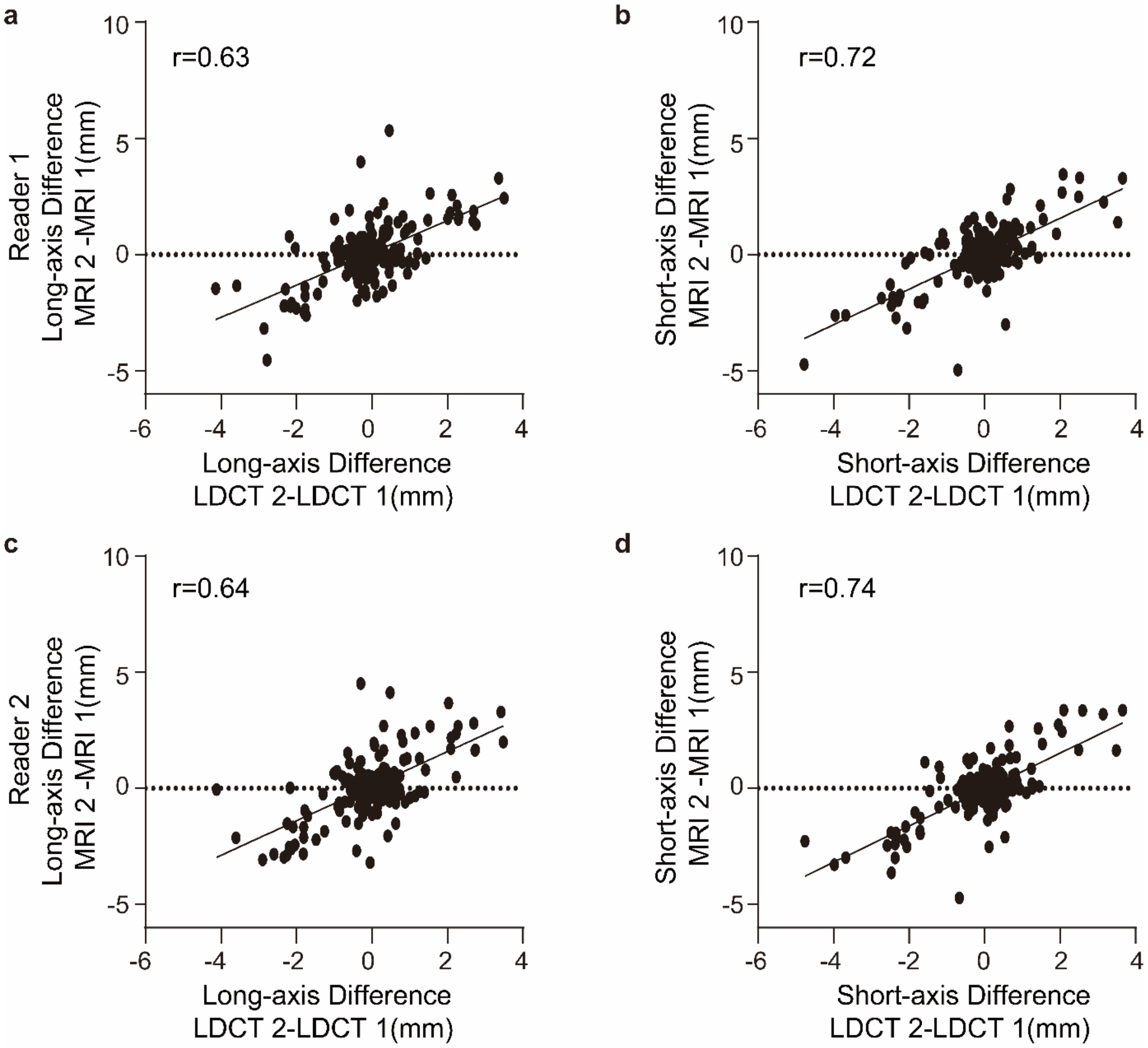

Supplemental Figure S3. Correlation of diameter change measured on LDCT with MRI.

Correlation of changes in long- and short-axis diameters from LDCT1 to LDCT2 with changes from MRI1 to MRI2 measured by reader 1 (a, b) and reader 2 (c, d).
